# Homoarginine- and Creatine-Dependent Gene Regulation in Murine Brains with l-Arginine:Glycine Amidinotransferase Deficiency

**DOI:** 10.3390/ijms21051865

**Published:** 2020-03-09

**Authors:** Märit Jensen, Christian Müller, Edzard Schwedhelm, Priyadharshini Arunachalam, Mathias Gelderblom, Tim Magnus, Christian Gerloff, Tanja Zeller, Chi-un Choe

**Affiliations:** 1 University Heart and Vascular Centre Hamburg, Clinic for Cardiology, University Medical Centre Hamburg-Eppendorf, 20246 Hamburg, Germany; m.jensen@uke.de (M.J.); ch.mueller@uke.de (C.M.); t.zeller@uke.de (T.Z.); 2 German Centre for Cardiovascular Research (DZHK e.V.), Partner Site Hamburg/Kiel/Lübeck, 20246 Hamburg, Germany; schwedhelm@uke.de; 3 Department of Neurology, University Medical Centre Hamburg-Eppendorf, 20246 Hamburg, Germany; apriyadharsh@gmail.com (P.A.); mgelderblom@uke.de (M.G.); t.magnus@uke.de (T.M.); gerloff@uke.de (C.G.); 4 Institute of Clinical Pharmacology and Toxicology, University Medical Centre Hamburg-Eppendorf, 20246 Hamburg, Germany

**Keywords:** AGAT, homoarginine, creatine, gene expression, microarray, cerebrovascular disease, ischemic stroke

## Abstract

l-arginine:glycine amidinotransferase (AGAT) and its metabolites homoarginine (hArg) and creatine have been linked to stroke pathology in both human and mouse studies. However, a comprehensive understanding of the underlying molecular mechanism is lacking. To investigate transcriptional changes in cerebral AGAT metabolism, we applied a transcriptome analysis in brains of wild-type (WT) mice compared to untreated AGAT-deficient (AGAT^−/−^) mice and AGAT^−/−^ mice with creatine or hArg supplementation. We identified significantly regulated genes between AGAT^−/−^ and WT mice in two independent cohorts of mice which can be linked to amino acid metabolism (*Ivd*, *Lcmt2*), creatine metabolism (*Slc6a8*), cerebral myelination (*Bcas1*) and neuronal excitability (*Kcnip3*). While *Ivd* and *Kcnip3* showed regulation by hArg supplementation, *Bcas1* and *Slc6a8* were creatine dependent. Additional regulated genes such as *Pla2g4e* and *Exd1* need further evaluation of their influence on cerebral function. Experimental stroke models showed a significant regulation of *Bcas1* and *Slc6a8*. Together, these results reveal that AGAT deficiency, hArg and creatine regulate gene expression in the brain, which may be critical in stroke pathology.

## 1. Introduction

l-arginine:glycine-amidinotransferase (AGAT; EC: 2.1.4.1) is the rate-limiting enzyme in creatine and l-homoarginine (hArg) synthesis. Both compounds have been linked to cerebrovascular function and disease, but the involved genes and pathways are not been completely uncovered.

The role of creatine for the brain has arisen from studies on creatine deficiency disorders (e.g., syndromes due to mutations affecting the *AGAT* gene) [1,2,3]. Patients showed altered brain function such as global developmental delay, intellectual disability and behavioral disorders. Cerebral creatine metabolism plays an essential role in GABAergic and glutamatergic signaling [4]. In experimental studies, creatine supplementation improved reperfusion and conferred neuroprotection in cerebral ischemia [5,6]. In humans, creatine supplementation enhanced cognitive performance and corticomotor excitability during oxygen deprivation [7].

Low plasma concentrations of hArg have been associated with etiologies and outcome after ischemic stroke [8,9]. The physiological role of hArg is not fully understood. Given its structural similarity to l-arginine, hArg can serve as an alternative substrate for nitric oxide synthase (NOS) and, in support of this, hArg levels have been associated with endothelial function [10]. Furthermore, hArg can competitively inhibit arginase and therefore increase l-arginine bioavailability and subsequently nitric oxide (NO) production [10]. Epidemiological studies have implied an involvement in atherosclerosis, as hArg levels were inversely associated with aortic wall thickness, aortic plaque burden and internal carotid artery stenosis [11,12,13]. Consistently, low hArg levels have been associated with stroke incidence, fatal strokes and outcome after stroke [8,9,11].

In humans, single-nucleotide polymorphisms (SNPs) within the *AGAT* gene are associated with altered hArg plasma concentrations [9,14]. Previously, we have shown that AGAT-deficient (AGAT^−/−^) mice with whole-body hArg and creatine deficiency revealed increased infarct sizes and aggravated neurological deficits after ischemic stroke. The supplementation with hArg, but not creatine, significantly reduced infarct sizes and improved outcome [9]. In addition to experimental stroke models, hArg supplementation proved protective in murine models of post-myocardial infarction heart failure, diabetic kidney disease, coronary artery disease and balloon-injured carotids [15,16,17,18]. However, data on the underlying molecular mechanisms and signal transduction pathways in the AGAT metabolism is still very limited.

In this study, we analyzed the global brain transcriptome of WT mice, untreated AGAT^−/−^ mice and AGAT^−/−^ mice supplemented with creatine (AGAT^−/−^Cr) or hArg (AGAT^−/−^hArg). The aim of our study was to identify potential pathways and regulated genes related to creatine or hArg supplementation. Furthermore, candidate genes were evaluated in an experimental stroke model in WT mice.

## 2. Results

### 2.1. Gene Expression Differences between WT and AGAT^−/−^ Mice in Brain Samples

**Figure 1:**
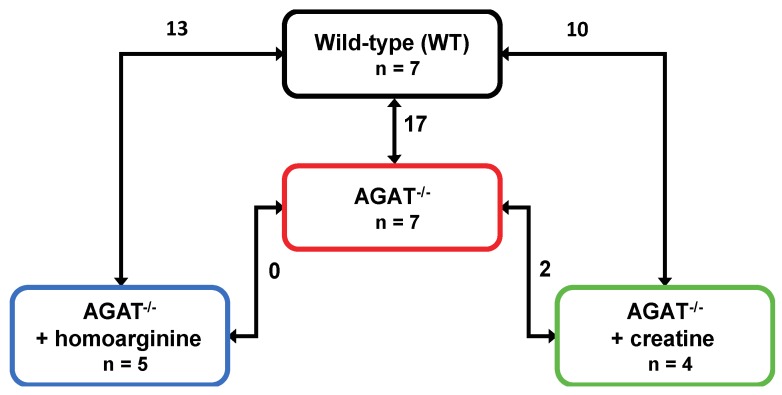
Number of differentially expressed genes in brains of WT, AGAT^−/−^, AGAT^−/−^hArg and AGAT^−/−^Cr mice. Transcriptome profiling was performed using the Affymetrix Mouse GeneChip 1.0 ST Array. Each line indicates the comparison of the respective two groups and the number of significantly regulated genes. Significance level: false discovery rate ≤ 0.05. Abbreviations: AGAT^−/−^, AGAT knock-out; n, number of animals.

**Figure 2:**
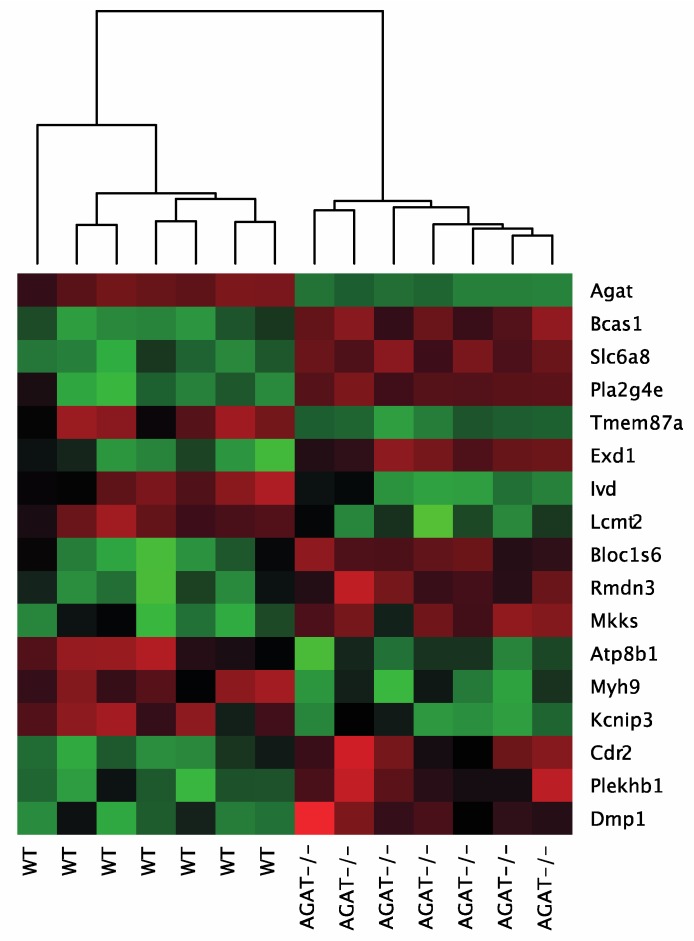
Heatmap of gene expression values depicting clustering of genes between WT and AGAT^−/−^ in brain tissue samples. The heatmap is based on the expression of mRNAs for the significant genes. Low to high expression is represented by a change of color from green to red, respectively. Abbreviations: AGAT^−/−^, AGAT knock-out mice; WT, wild-type mice.

We performed a global transcriptome analysis of left hemisphere tissue of WT, AGAT^−/−^, AGAT^−/−^hArg and AGAT^−/−^Cr mice. The number of differentially expressed genes was evaluated for each comparison (Figure 1). Comparison of WT and AGAT^−/−^ mice revealed 17 significantly regulated genes (FDR ≤ 0.05; see Table 1 and Figure 2). Of these 17 genes, eight genes were validated in an independent cohort of mice, i.e., *Agat*, *Bcas1*, *Slc6a8*, *Pla2g4e*, *Exd1*, *Ivd*, *Lcmt2* and *Kcnip3*. Effect sizes and directions were largely concordant between the two studies.

### 2.2. Creatine- and hArg-Dependent Gene Regulation in the brain

Creatine and hArg supplementation have beneficial effects in AGAT^−/−^ mice on cognitive function and cerebral ischemia, respectively [9,19]. Therefore, we aimed to evaluate gene regulation in brains of AGAT^−/−^ mice after either creatine or hArg supplementation. We identified ten genes that were significantly different between WT and AGAT^−/−^Cr mice, i.e., *Agat*, *Lcmt2*, *Rassf2*, *Mkks*, *Ivd*, *Reps2*, *Magee2*, *Prokr2*, *Tmem47* and *Pla2g4e* (FDR ≤ 0.05; Table 2). The comparison of AGAT^−/−^ with AGAT^−/−^Cr and AGAT^−/−^hArg mice did not elicit a relevant number of significantly regulated genes using genome-wide approach with fully adjusted significance levels. To identify more distinct differences of creatine and hArg supplementation in AGAT^−/−^ mice, genes were selected by comparison of WT and AGAT^−/−^Cr to identify potential creatine-regulated genes and WT and AGAT^−/−^hArg mice to identify potential hArg-regulated genes. A regulation is indicated by a restoration of expression levels towards WT levels in supplemented animals. Creatine supplementation in AGAT^−/−^ mice leads to a normalization of twelve genes that were significantly regulated within the comparison of WT and AGAT^−/−^ mice, i.e., *Bcas1*, *Slc6a8*, *Tmem87a*, *Exd1*, *Bloc1s6*, *Rmdn3*, *Atp8b1*, *Myh9*, *Kcnip3*, *Cdr2*, *Plekhb1* and *Dmp1*. In AGAT^−/−^hArg mice, 13 genes were significantly regulated compared to their expression levels in WT mice, i.e., *Agat*, *Bcas1*, *Slc6a8*, *Pla2g4e*, *Tmem87a*, *Exd1*, *Lcmt2*, *Ivd*, *Mkks*, *Cd59a*, *B2m*, *Prokr2* and *Nfkbia* (Table 3). The expression of eight genes was normalized to WT levels in AGAT^−/−^hArg mice, i.e., *Bloc1s6*, *Rmdn3*, *Atp8b1*, *Myh9*, *Kcnip3*, *Cdr2*, *Plekhb1* and *Dmp1*.

### 2.3. Selection and Validation of Cerebrovascular Candidate Genes

Among the differentially expressed genes between WT and AGAT^−/−^ mice, cerebrovascular candidate genes were chosen based on positive validation in the replication cohort and known association with cerebral metabolism and pathologies (i.e., *Bcas1*, *Slc6a8, Ivd*, *Lcmt2*, *Kcnip3*, *Exd1* and *Pla2g4e*).

The second most strongly regulated gene, breast carcinoma amplified sequence 1 (*Bcas1*, *p* = 9.07 × 10^−11^) may influence cerebral myelination [20]. The solute carrier family 6 (neurotransmitter transporter, creatine), member 8 (*Slc6a8*, *p* = 1.46 × 10^−9^) is a specific plasma membrane transporter that further enables cells to incorporate creatine and take up the precursor guanidinoacetate, which directly contributes to creatine biosynthesis [4]. Isovaleryl coenzyme A dehydrogenase (*Ivd*, *p* = 1.45 × 10^−6^) and leucine carboxyl methyltransferase 2 (*Lcmt2*, *p* = 1.98 × 10^−6^) were both downregulated in AGAT^-/-^ mice. While the IVD enzyme is involved in L-leucine degradation, the LCMT2 enzyme may methylate the carboxyl group of leucine residues to form alpha-leucine ester residues. This finding is of interest as branched-chain amino acids, particularly leucine, play an important role in glutamate synthesis. Kv channel interacting protein 3, calsenilin (*Kcnip3*, *p* = 1.48 × 10^−5^) encodes a member of proteins interacting with voltage-gated potassium (Kv) channels. Members of this family are small calcium binding and integral subunit components of native Kv4 channel complexes that may regulate neuronal excitability [21]. Phospholipase A2, group IVE (*Pla2g4e*, *p* = 1.9 × 10^−8^) and exonuclease 3’−5’ domain containing 1 (*Exd1*, *p* = 5.08 × 10^−7^), which were both upregulated in AGAT^−/−^ mice, have not been associated with cerebrovascular function before.

**Figure 3:**
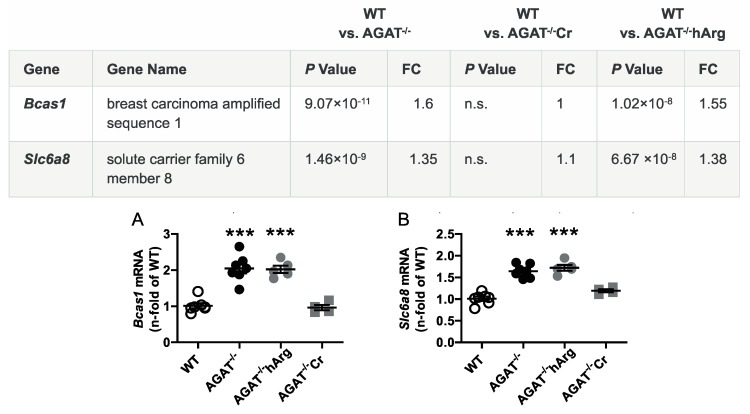
Validation of potential creatine-dependent candidate genes by qPCR. Relative mRNA expression of *Bcas1* (**A**) and *Slc6a8* (**B**) in the brain. Each data point represents an individual mouse. *** *p* < 0.001 versus WT. WT (*n* = 7), AGAT^−/−^ (*n* = 7), AGAT^−/−^hArg (*n* = 5), AGAT^−/−^Cr (*n* = 4). The table shows the respective microarray data. Abbreviations: FC, fold change; n.s., not significant.

**Figure 4:**
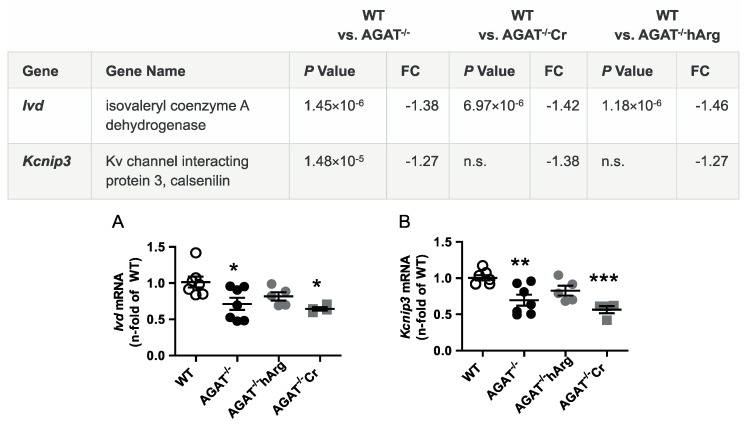
Validation of potentially hArg-dependent candidate genes by qPCR. Relative mRNA expression of *Ivd* (**A**) and *Kcnip3* (**B**) in the brain. Each data point represents an individual mouse. * *p* < 0.05, ** *p* < 0.01 and *** *p* < 0.001 versus WT. WT (*n* = 7), AGAT^−/−^ (*n* = 7), AGAT^−/−^hArg (*n* = 5), AGAT^−/−^Cr (*n* = 4). The table shows the respective microarray data. Abbreviations: FC, fold change; n.s., not significant.

**Figure 5:**
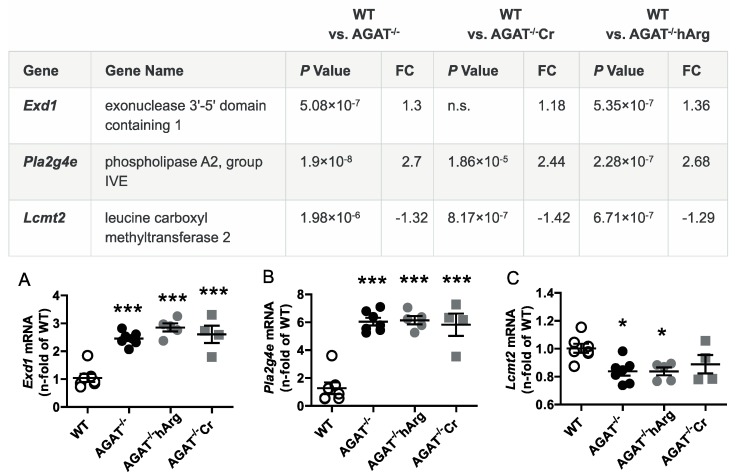
Validation of potential AGAT-dependent candidate genes by qPCR. Relative mRNA expression of *Exd1* (**A**), *Pla2g4e* (**B**) and *Lcmt2* (**C**) in the brain. Each data point represents an individual mouse. * *p* < 0.05 and *** *p* < 0.001 versus WT. WT (*n* = 7), AGAT^−/−^ (*n* = 7), AGAT^−/−^hArg (*n* = 5), AGAT^−/−^Cr (*n* = 4). The table shows the respective microarray data. Abbreviations: FC, fold change; n.s., not significant.

All selected candidate genes were validated by qPCR. As expected from the transcriptome analysis, we found that mRNA expression levels of both *Bcas1* and *Slc6a8* were restored to WT levels in AGAT^-/-^Cr mice indicating a creatine-dependent regulation (Figure 3). The supplementation of hArg normalized *Kcnip3* mRNA levels towards WT levels. In addition, qPCR analysis showed that *Ivd* expression may be dependent on hArg, as the comparison of WT and AGAT^−/−^hArg revealed no significant difference of *Ivd* levels between both groups (Figure 4). Our results revealed that mRNA expression of *Exd1*, *Pla2g4e* and *Lcmt2* was significantly altered in AGAT^−/−^ mice, however, neither creatine nor hArg supplementation normalized the expression of these genes towards WT levels (Figure 5).

### 2.4. Confirmation of Candidate Gene Expression in a Mouse Model of Ischemic Stroke

**Figure 6:**
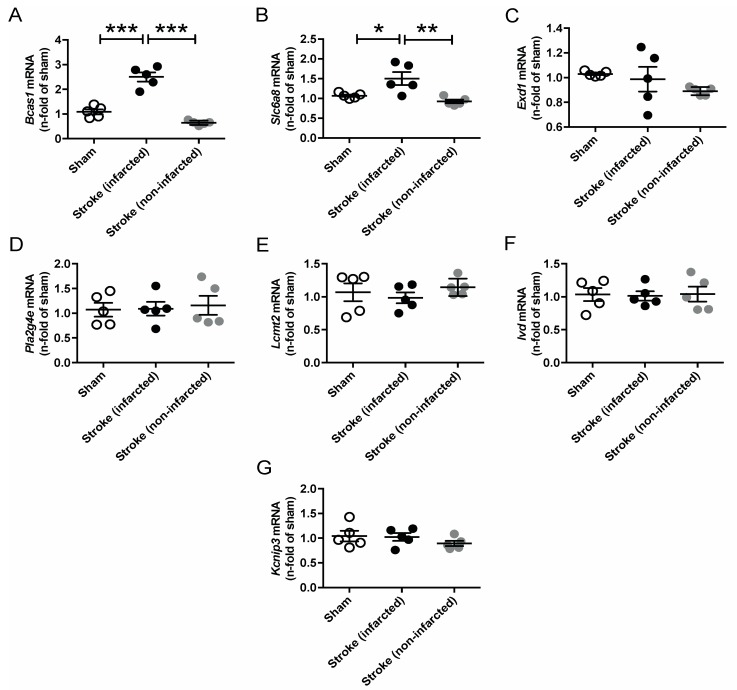
Relative mRNA expression of *Bcas1* (**A**), *Slc6a8* (**B**), *Exd1* (**C**), *Pla2g4e* (**D**), *Lcmt2* (**E**), *Ivd* (**F**) and *Kcnip3* (**G**) candidate genes 3 days after tMCAO in C57BL/6J WT mice. Each data point represents an individual mouse. * *p* < 0.05, ** *p* < 0.01 and *** *p* < 0.001 versus WT. Sham (*n* = 5), stroke (infarcted area, *n* = 5), stroke (noninfarcted area, *n* = 5).

Since AGAT/hArg/creatine metabolism has been linked to ischemic stroke in both humans and mice, we performed temporary middle cerebral artery occlusion (tMCAO) in C57BL/6J WT mice to assess the regulation of our candidate genes in response to cerebrovascular disease [8,9]. The analysis revealed a significant upregulation of *Bcas1* and *Slc6a8* after ischemic stroke (Figure 6). Expression levels of all other candidate genes (i.e., *Exd1*, *Pla2g4e*, *Lcmt2*, *Ivd*, *Kcnip3*) were not altered in the infarcted or noninfarcted brain.

### 2.5. Amino acid Analysis in Brain Tissue of AGAT^−/−^ Mice

**Figure 7:**
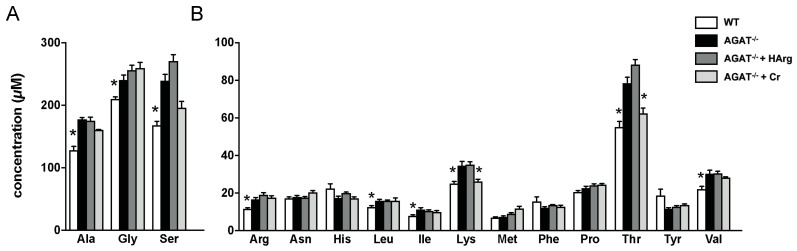
Amino acid analysis in brain tissue of WT, AGAT^−/−^, AGAT^−/−^Cr and AGAT^−/−^hArg mice. (**A**) Amino acids with high concentrations. (**B**) Amino acids with lower concentrations. * *p* < 0.05 versus AGAT^−/−^. Mann–Whitney U-test. Adjustment for multiple testing by Benjamini–Hochberg method. False discovery rate ≤ 0.05. Amino acid abbreviations are in three letter code.

Given that certain candidate genes (e.g., *Ivd* and *Lcmt2*) are involved in amino acid metabolism, we have analyzed amino acid profiles in brain tissue of WT, AGAT^−/−^, AGAT^−/−^Cr and AGAT^−/−^hArg mice. Tandem mass spectrometry revealed increased concentrations of alanine, glycine, serine, arginine, leucine, isoleucine, lysine, threonine and valine in AGAT^−/−^ mice compared with WT mice (Figure 7). Comparing AGAT^−/−^ and AGAT^−/−^Cr mice, concentrations of lysine and threonine were reduced towards WT levels indicating a normalization of metabolite levels by creatine. 

## 3. Discussion

In this work, we demonstrate that: (1) eight validated genes are differentially expressed in AGAT^-/-^ compared with WT mice; (2) altered *Bcas1* and *Slc6a8* expression is creatine-dependent; (3) *Kcnip3* and *Ivd* expression levels are hArg-dependent; and (4) expression of *Bcas1* and *Slc6a8* is increased after stroke. In total, after excluding *Agat*, we identified seven candidates that have been validated by microarray analysis in an independent cohort of mice and by qPCR analysis. Among those seven genes, five have been discussed in regard to cerebrovascular function in literature. These genes can be linked to cerebral myelination (*Bcas1*) [20], creatine metabolism (*Slc6a8*) [22,23], neuronal excitability (*Kcnip3*) [24,25] and amino acid metabolism (*Ivd*, *Lcmt2*) [26]. *Bcas1* and *Slc6a8* revealed a creatine-dependent gene regulation, whereas *Kcnip3* and *Ivd* seem to be hArg-dependent.

*Bcas1*, encoding for the protein BCAS1, a basic protein abundant in the brain, was significantly upregulated in AGAT^−/−^ mice. Previous research showed that BCAS1 was expressed specifically in oligodendrocytes and Schwann cells and that its expression level was decreased in demyelinating processes. Moreover, the loss of BCAS1 specifically induced hypomyelination and expression of inflammation-related genes in the brain [20]. In line with previous studies, we found increased *Bcas1* levels after stroke which are likely to represent a compensatory mechanism of oligodendrocyte proliferation after hypoxia and energy deficiency [27]. *Bcas1* levels were increased in AGAT^-/-^ mice and correlate with cognitive impairment [19]. Both cognitive function and *Bcas1* levels in AGAT^-/-^ mice normalized with creatine supplementation. Therefore, *Bcas1* expression and cognitive function in AGAT^-/-^ mice could be linked with each other. After synthesis in kidney and liver, creatine is transported to organs of high fluctuating energy demand, i.e., skeletal muscle, heart and brain. *Slc6a8* encodes the creatine transporter, which mediates the active uptake. Mouse models of creatine transporter deficiency revealed impaired cognitive and motor function [22,23]. In AGAT^−/−^ mice, we found an upregulation which is likely to be a compensatory mechanism due to creatine deficiency. Consistently, creatine supplementation normalized *Slc6a8* expression levels in AGAT^−/−^ mice (indicated by no significant regulation comparing WT and AGAT^−/−^Cr). Similarly, *Slc6a8* expression was strongly increased in skeletal muscle of AGAT^−/−^ mice [28]. Of note, we observed an upregulation of *Slc6a8* mRNA in the infarcted as compared with the contralateral hemisphere indicating local creatine deficiency after injury.

Whereas altered expression levels of *Bcas1* and *Slc6a8* were normalized with creatine supplementation, *Kcnip3* and *Ivd* gene expression approached WT levels upon hArg supplementation. KCNIP3, also known as calsenilin, is a neuronal calcium-binding protein that has been shown to have multiple functions in the cells, including regulating the intracellular concentration of calcium, the binding and modulation of the Alzheimer’s-disease-related protein presenilin and controlling of multiple signaling pathways as a second messenger [24,25]. In line with the role of KCNIP3 for cellular function, diseases associated with *KCNIP3* gene mutations include Alzheimer’s disease [21]. *Kcnip3* mRNA was downregulated in AGAT^-/-^ mice, which revealed severe cognitive impairment. Although creatine supplementation improved motor learning and memory function (i.e., rotarod, water maze), *Kcnip3* expression levels were increased after hArg, but not creatine alone [19]. *Ivd* expression levels were also decreased in AGAT^−/−^ mice. The IVD enzyme plays a role in L-leucine degradation. This finding is of interest as branched-chain amino acids, particularly leucine, play an important role in glutamate synthesis [26]. As *Ivd* influences leucine metabolism, hArg may be involved in the regulation of branched-chain amino acids. Metabolomic analysis of AGAT^-/-^ brain tissue revealed a significantly altered amino acid metabolism, including increased leucine levels. In light of transcriptomic and metabolomic analyses, either increased leucine levels alter *Ivd* gene expression or vice versa under conditions of AGAT deficiency. Although hArg supplementation normalized *Ivd* expression levels, leucine concentrations did not normalize with hArg supplementation. Therefore, the interplay between hArg, Ivd and amino acid metabolism seems much more complex. Analysis of gene expression and protein metabolism in specific neuronal and glial subpopulations would potentially shed more light on this complex interaction.

Given the severe phenotype of AGAT^−/−^ mice, it is surprising that only eight validated genes in the brain were differentially regulated between AGAT^−/−^ and WT mice [29,30]. Previously, transcriptomic analyses of AGAT^−/−^ skeletal muscle uncovered more than 100 genes, involved in glucose, pyruvate and one-carbon metabolism [28]. In contrast to the present study, the previous analysis in skeletal muscle did not include a validation in a second independent cohort. Creatine supplementation completely normalized the metabolic phenotype of AGAT^−/−^ mice (i.e., weight, glucose and lipid metabolism), whereas only hArg supplementation improved post-myocardial infarction heart failure, diabetic kidney damage, systolic function in a model of coronary artery disease and reduced neointimal hyperplasia in balloon-injured rat carotids [15,17,18,29]. Interestingly, hArg normalized a number of cardiac parameters, which are highly calcium-dependent despite unaltered intracellular calcium levels [15,31]. In this light, increased gene expression levels of the neuronal calcium-binding protein KCNIP3 could similarly influence neuronal excitability and signaling. Although hArg proved protective in disease models of various organ failures (i.e., kidney, heart, brain), calcium signaling and potassium channel physiology represent common mechanisms in all pathologies.

The current experimental design has limitations. First, an analysis of different cell types (i.e., glia and neurons) and subpopulations was not possible, as whole hemispheres were used in this study. Therefore, the analysis did not detect specific changes and we were not able to differentiate between different cell types. Additionally, other regions that determine the cerebral phenotype of AGAT^−/−^ mice have been systematically overlooked. Therefore, future studies should include a specific analysis of different brain areas and cell types. Second, we did not analyze protein levels of genes with altered expression levels. Additional analysis and detailed assessment of target genes identified in our transcriptome analysis such as protein and/or metabolome analysis have to be performed. Finally, this study did not comprise an AGAT^-/-^ disease model such as an experimental stroke model. The analysis of gene expression in an AGAT^-/-^ disease model would directly link the stroke phenotype to underlying molecular mechanisms on the transcriptome level. The current study does, however, demonstrate that the whole-brain approach identified important candidate genes in the AGAT metabolism.

In summary, our analysis identified differentially regulated genes in brains of AGAT^-/-^ mice dependent on creatine or hArg. These data should enable the generation of new hypotheses to gain more insights into potential mechanisms of hArg.

## 4. Materials and Methods

### 4.1. Care and Trearment of Mice

AGAT^−/−^ mice were generated as previously described [29]. Mice used in this study were obtained from heterozygous breeding after backcrossing to a C57BL/6J genetic background for at least six generations. All analyzed animals were littermates. The mice (<5 per cage) were kept in standard cages under a 12 h:12 h light:dark cycle and constant temperature and humidity, receiving standard food and water ad libitum. The 4-week-long supplementation with hArg was achieved via osmotic mini pumps [9]. Creatine supplementation was achieved by addition of 1% creatine to chow (Ssniff) as previously described [29]. All experimental procedures were approved by the respective local animal ethics committees (Behörde für Gesundheit und Verbraucherschutz Hamburg, approval no. 110/10, approval date day month yearFebruary 15, 2011) and investigations applied to the animal model were in accordance with the guidelines for the care and use of laboratory animals published by the NIH (Publication No. 85–23, revised 1985).

### 4.2. Tissue Collection and Preparation

Tissue collection and preparation were performed as previously described [29]. Briefly, mice were anesthetized with 2–3% isoflurane in 100% oxygen. Left and right hemisphere were extracted and shock frozen in liquid nitrogen for storage at −80 °C. Prior to use, frozen tissue was powdered with a steel mortar and pestle in liquid nitrogen.

### 4.3. RNA Isolation from Murine Tissue

RNA isolation was performed using QIAzol lysis reagent. Briefly, frozen tissue powder was minced in QIAzol and further disrupted using a pellet pestle. To extract RNA, chloroform was added, mixed, and centrifuged. The aqueous phase containing the RNA was collected and isopropanol was added. For precipitation, the RNA solution was centrifuged 15 min at 4 °C at high speed. After washing with 80% ethanol twice, the RNA pellet was dissolved in nuclease-free water. RNA concentration was determined by measuring absorbance at 260 nm using Nanodrop. RNA was stored at −80 °C until utilization.

### 4.4. In vivo Model of Ischemic Stroke in Mice

Temporary middle cerebral artery occlusion (tMCAO) in C57BL/6J WT mice was achieved by using the intraluminal filament method (6-0 nylon) for one hour as described previously [32]. The control mice group underwent sham surgery and all animals were sacrificed three days after reperfusion.

### 4.5. Gene Expression Analysis by Microarray

Total RNA was prepared in four groups of mice: WT (*n* = 7), AGAT^−/−^ (*n* = 7), AGAT^−/−^hArg (*n* = 5) and AGAT^−/−^Cr (*n* = 4) mice. RNA integrity was assessed on a 2100 Agilent Bioanalyzer (Agilent Technologies, Waldbronn, Germany). The Affymetrix Mouse GeneChip ST 1.0 Array was used to assess the gene expression profile. Briefly, cRNA synthesis, labelling, fragmentation, array hybridization, washing and staining, along with microarray scanning (Affymetrix GeneChip 3000 scanner), was performed according to manufacturer’s instructions of the Ambion WT Expression Kit and the Affymetrix GeneChip WT Terminal Labelling and Hybridization Kit with an input of 250 ng high-quality RNA (RIN > 8).

### 4.6. Reverse Transcription and Quantitative Polymerase Chain Reaction (qPCR)

Reverse transcription and qPCR were performed as previously described [33]. One microgram of total RNA was reverse transcribed using the High Capacity Kit (Life Technologies, Carlsbad, CA, USA). For reverse transcription, samples were incubated for 2 h at 37 °C followed by an inactivation step of 5 min at 85 °C. Finally, cDNA was diluted in water to a final concentration of 5 ng/µL. The relative quantification of mRNA levels was carried out on a 7900 TaqMan system (Applied Biosystems, Foster City, CA, USA). To assess mRNA expression of target genes, real-time PCR was performed using 5 µL of the gene expression master mix (Thermo Fisher, Waltham, MA, USA) and 0.5 µL of the gene expression assay (Table 4). Each gene expression assay includes forward and reverse primers as well as the FAM-labelled probe. As template, 2 µL of cDNA was used in a final volume of 10 μL for detection. Each sample was analyzed in duplicates and normalized to 18S rRNA as endogenous control.

### 4.7. Amino acid Analysis

Amino acid analysis of brain tissue was performed with tandem mass spectrometry using the Biocrates AbsoluteIDQ p180 Kit (Biocrates Life Sciences AG, Innsbruck, Austria). The protocols of sample processing and metabolite extraction from brain were adapted by calculating the volume of the solvent used for extracting metabolites on the basis of sample weight, as described elsewhere (Application note 1004-1, Biocrates, Bogumil, R. 2009; available online: http://www.biocrates.com/images/stories/pdf/biocrates_appl.note_1004-1.pdf).

### 4.8. Bioinformatics Analysis

Differential gene expression analyses of murine transcriptomes were performed using the statistical language R and R/Bioconductor packages xps and limma. Microarrays were preprocessed using the package xps. The rma function was used for background correction and normalization in order to reduce variation between arrays. The detection above background (DABG) was calculated for all genes and samples, and only genes with a DABG *p* value < 0.01 in at least two samples per group were kept for further analysis. The moderated t-test function eBayes from the limma package was used to calculate differential gene expression between groups. The false discovery rate (FDR)-based Benjamini–Hochberg method was used to account for multiple tests, and the significance level for differentially expressed genes was set to a FDR ≤ 0.05.

### 4.9. Metabolome Analysis

Metabolites with concentrations above the lower detection limit (LOD) were considered present. Only metabolites present in at least three samples from one tissue were used for further analysis. Statistical differences were computed for each metabolite by Mann–Whitney U-test. To account for multiple testing, the FDR-based Benjamini–Hochberg method was used. Metabolites with a FDR ≤ 0.05 were considered significantly different.

### 4.10. Statistical Analysis

Values are expressed as mean ± SEM. The mRNA levels were quantified according to the 2^−∆∆Ct^ method by Livak and Schmittgen [34]. For comparison of multiple groups, one-way ANOVA with Bonferroni post-hoc test was used. Differences were considered statistically significant at a value of *p* ≤ 0.05. All calculations were performed using Graph Pad Prism 7.

## Figures and Tables

**Table 1:** Differentially expressed genes between WT and AGAT^−/−^ mice in brain tissue. *p* values and fold changes (FC) are given for the discovery cohort and the validation cohort. False discovery rate ≤ 0.05. Significantly regulated genes in both cohorts are written in bold. Abbreviations: b.d.l., below detection limit.

Gene	Gene Name	Discovery Cohort		Validation Cohort	
		*p* Value	FC	*p* Value	FC
***Agat***	**L-arginine:glycine amidinotransferase**	**4.32 × 10^−16^**	**−2.91**	**6.95 × 10^−18^**	**−3.31**
***Bcas1***	**breast carcinoma amplified sequence 1**	**9.07 × 10^−11^**	**1.6**	**2.68 × 10^−8^**	**1.45**
***Slc6a8***	**solute carrier family 6 (neurotransmitter transporter, creatine), member 8**	**1.46 × 10^−9^**	**1.35**	**3.41 × 10^−9^**	**1.31**
***Pla2g4e***	**phospholipase A2, group IVE**	**1.9 × 10^−8^**	**2.7**	**2.85 × 10^−16^**	**4.33**
*Tmem87a*	transmembrane protein 87A	1.7 × 10^−7^	−1.3	1.81 × 10^−1^	1.04
***Exd1***	**exonuclease 3’−5’ domain containing 1**	**5.08 × 10^−7^**	**1.3**	**4 × 10^−8^**	**1.41**
***Ivd***	**isovaleryl coenzyme A dehydrogenase**	**1.45 × 10^−6^**	**−1.38**	**1.18 × 10^−10^**	**−1.39**
***Lcmt2***	**leucine carboxyl methyltransferase 2**	**1.98 × 10^−6^**	**−1.32**	**3.5 × 10^−6^**	**−1.23**
*Bloc1s6*	biogenesis of organelles complex-1, subunit 6, pallidin	2.61 × 10^−6^	1.37	b.d.l.	-
*Rmdn3*	regulator of microtubule dynamics 3	3.02 × 10^−6^	1.27	b.d.l.	-
*Mkks*	McKusick–Kaufman syndrome	3.8 × 10^−6^	1.42	3.3 × 10^−1^	1.04
*Atp8b1*	ATPase, class I, type 8B, member 1	1.02 × 10^−5^	−1.27	4.84 × 10^−3^	−1.14
*Myh9*	myosin, heavy polypeptide 9, nonmuscle	1.19 × 10^−5^	−1.21	1.84 × 10^−2^	−1.07
***Kcnip3***	**Kv channel interacting protein 3, calsenilin**	**1.48 × 10^−5^**	**−1.27**	**4.05 × 10^−6^**	**−1.17**
*Cdr2*	cerebellar degeneration-related 2	2 × 10^−5^	1.2	1.32 × 10^−2^	1.1
*Plekhb1*	pleckstrin homology domain containing, family B (evectins) member 1	2.7 × 10^−5^	1.19	2.05 × 10^−3^	1.1
*Dmp1*	dentin matrix protein 1	3.59 × 10^−5^	1.23	8.41 × 10^−2^	−1.05

**Table 2:** Differentially expressed genes between WT and AGAT^−/−^Cr mice in brain tissue. False discovery rate ≤ 0.05. Abbreviations: FC, fold change.

Gene	Gene Name	*p* Value	FC
*Agat*	l-arginine:glycine amidinotransferase	6.64 × 10^−13^	−2.85
*Lcmt2*	leucine carboxyl methyltransferase 2	8.17 × 10^−7^	−1.42
*Rassf2*	Ras association (RalGDS/AF-6) domain family member 2	8.22 × 10^−7^	−1.38
*Mkks*	McKusick–Kaufman syndrome	1.1 × 10^−6^	1.54
*Ivd*	isovaleryl coenzyme A dehydrogenase	6.97 × 10^−6^	−1.42
*Reps2*	RALBP1 associated Eps domain containing protein 2	8.94 × 10^−6^	−1.3
*Magee2*	melanoma antigen, family E, 2	1.03 × 10^−5^	−1.37
*Prokr2*	prokineticin receptor 2	1.04 × 10^−5^	−1.31
*Tmem47*	transmembrane protein 47	1.57 × 10^−5^	−1.31
*Pla2g4e*	phospholipase A2, group IVE	1.86 × 10^−5^	2.44

**Table 3:** Differentially expressed genes between WT and AGAT^-/-^hArg mice in brain tissue. False discovery rate ≤ 0.05. Abbreviations: FC, fold change.

Gene	Gene Name	*p* Value	FC
*Agat*	l-arginine:glycine amidinotransferase	2.05 × 10^−14^	−2.91
*Bcas1*	breast carcinoma amplified sequence 1	1.02 × 10^−8^	1.55
*Slc6a8*	solute carrier family 6 (neurotransmitter transporter, creatine), member 8	6.67 × 10^−8^	1.38
*Pla2g4e*	phospholipase A2, group IVE	2.28 × 10^−7^	2.68
*Tmem87a*	transmembrane protein 87A	2.52 × 10^−7^	−1.35
*Exd1*	exonuclease 3’−5’ domain containing 1	5.35 × 10^−7^	1.36
*Lcmt2*	leucine carboxyl methyltransferase 2	6.71 × 10^−7^	−1.29
*Ivd*	isovaleryl coenzyme A dehydrogenase	1.18 × 10^−6^	−1.46
*Mkks*	McKusick–Kaufman syndrome	7.82 × 10^−6^	1.36
*Cd59a*	CD59a antigen	1.09 × 10^−5^	1.35
*B2m*	beta-2 microglobulin	1.52 × 10^−5^	−1.35
*Prokr2*	prokineticin receptor 2	1.59 × 10^−5^	−1.31
*Nfkbia*	nuclear factor of kappa light polypeptide gene enhancer in B cells inhibitor, alpha	2.2 × 10^−5^	1.37

**Table 4:** Gene expression assays used for qPCR.

Gene	Assay ID
*Bcas1*	Mm00659626_m1
*Slc6a8*	Mm00506023_m1
*Ivd*	Mm00498171_m1
*Lcmt2*	Mm03647220_s1
*Kcnip3*	Mm01339777_m1
*Pla2g4e*	Mm00625711_m1
*Exd1*	Mm00556180_m1
